# CrossHybDetector: detection of cross-hybridization events in DNA microarray experiments

**DOI:** 10.1186/1471-2105-9-485

**Published:** 2008-11-17

**Authors:** Paolo Uva, Emanuele de Rinaldis

**Affiliations:** 1 Istituto di Ricerche di Biologia Molecolare, Merck Research Laboratories, 00040 Pomezia, Rome, Italy

## Abstract

**Background:**

DNA microarrays contain thousands of different probe sequences represented on their surface. These are designed in such a way that potential cross-hybridization reactions with non-target sequences are minimized. However, given the large number of probes, the occurrence of cross hybridization events cannot be excluded. This problem can dramatically affect the data quality and cause false positive/false negative results.

**Results:**

*CrossHybDetector *is a software package aimed at the identification of cross-hybridization events occurred during individual array hybridization, by using the probe sequences and the array intensity values. As output, the software provides the user with a list of array spots potentially 'corrupted' and their associated p-values calculated by Monte Carlo simulations. Graphical plots are also generated, which provide a visual and global overview of the quality of the microarray experiment with respect to cross-hybridization issues.

**Conclusion:**

*CrossHybDetector *is implemented as a package for the statistical computing environment *R *and is freely available under the LGPL license within the CRAN project.

## Background

Expression microrrays are used in a wide range of applications to simultaneously monitor the relative abundance of thousands of target sequences. A basic requirement of array probes is that of having low reciprocal similarity in order to reduce the likelihood of cross-hybridization effects. On the other hand, the permissible sequence 'dissimilarity' between probes is constrained by the requirement that probes have to share similar temperatures of annealing. This guarantees that all target sequences correctly hybridize with their probes in the same experimental conditions. Thus, the optimal array probe design is a trade-off solution between probe Tm similarities and probe sequence 'dissimilarities'. As a consequence, the greater the number of probes spotted on the array, the greater the chance of cross-hybridization events occurring. In this case the signal intensity measured by a chip spot carrying a given probe is affected by the unspecific binding of an off-target sequence similar to the target sequence. This effect, if not detected, can result in a number of false positives signals on the array. Even in the case of optimal probe design, suboptimal experimental conditions may favor cross-hybridization over specific binding [1]. This issue can be particularly relevant for customized microarray designs. In fact, hybridization protocols of standard commercial platforms are optimized and rigorous quality controls are carried out before the microarray platform gets deployed [2,3]. On the contrary, customized arrays are more prone to be affected by cross-hybridization or other issues related to the novel probe design. As a consequence, the set up of ad-hoc quality controls procedures is a crucial pre-requisite to improve data quality. *CrossHybDetector *identifies probes highly similar and checks for 'suspicious' spot intensity patterns based on the outcome of a single microarray experiment. A p-value expressing the likelihood of the pattern occurring by chance is calculated for each probe using Monte Carlo simulations. In addition, a global 'cross-hybridization quality control' parameter is provided in output and plots are generated which allow a visual overview of the cross-hybridization events in the microarray experiment. Here we present the *CrossHybDetector *software and, as a proof of concept, we illustrate two case studies in which the software and the underlying methodology have been successfully applied to detect cross-hybridization events.

## Implementation

*CrossHybDetector *is implemented as a package within the statistical computing environment R [4]. Functions of *marray *and *methods R *packages are internally utilized and are required by *CrossHybDetector *to work.

### Data formats

*CrossHybDetector *algorithm uses as input data i) the array probe sequences, ii) the spot intensities and array layout, iii) the spot type information (i.e. for each spot, whether it is "standard probe", "negative control", "spike-in"). This information is respectively contained into three separated text files. Exemplary analysis and related input files are submitted as  supplemental materials (Additional files 1 and 2).

### Algorithm

A cross-hybridization event takes place when a target sequence not only hybridizes to its related spot(s) on the chip, but also 'corrupts' chip spots carrying probes with similar sequences. The degree of the cross-hybridization effect is proportional to the sequence similarity between probes and to the relative abundance of the off-target sequence versus the target sequence. As a consequence, abundant target sequences can generate high signal intensities on their related chip spots as well as 'pushing ahead' the intensity values of spots carrying similar probes. The algorithm implemented in *CrossHybDetector *works as follows:

1. The probes with the highest intensities are selected as follows: probes with intensities higher than the saturation value (default = 65535) OR probes with intensity higher than a z-score threshold (default = 3). Among the two generated probe list, the largest one is selected. These probes are more prone to cause detectable cross-hybridization events on probes with similar sequences. In principle even low abundant targets could cross-hybridize to non specific probes on the chip, but this would have a minor impact on the final readout. For this reason these probes are not considered by default. However, the default threshold values can be modified by the user to extend the analysis to the probes with lower intensities.

2. Each of the selected probes is aligned against all the others. For each pairwise alignment the similarity between sequences is expressed as the Smith-Waterman (SW) score [5].

Among the available measures of pairwise sequence similarity (i.e. percent identity, longest common stretch) [6,7], we decided to adopt the SW score. This metric can be rapidly computed and is reported to be highly correlated (r = 0.98, p-value p < 10^-165^) with the best univariate predictor of cross-hybridization ("most contiguous base pairs between probe and target sequences") [7].

In the presence of a particular composition of the probes spotted on the array, alternative methods to compute the pairwise sequence similarity can be easily plugged in.

3. All the probes similar in sequence to probe *i *are identified by selecting all the alignments with a ΔSW score below a user-defined threshold. ΔSW is computed as:

ΔSW_i,k _= SW_i,i _- SW_i,k_

where SW_i,i _is the score of the alignment of probe_i _vs. probe_i _('perfect' pairing) and SW_i,k _is the score of the aligment of probe_i _vs probe_k _('imperfect' pairing).

4. Monte Carlo simulations: the sum of intensities for each subset of probes selected in 3 is compared against the distribution of the sums obtained from the random sampling of an equal number of probes on the array (10,000 samplings by default). A p-value is therefore calculated for each subset of probes as the fraction of the random samplings having a sum of intensities higher than the observed value, and corrected for multiple testing using the FDR procedure [8]. Probe subsets with p-value below a user-defined threshold (0.01 by default) are assessed as being composed of probes affected by unspecific binding. These probes are flagged as *corrupted*. The probe corresponding to the target sequence causing cross-hybridization is instead flagged as *corruptor*. A *corruptor/corrupted *probe pair is therefore composed by a *corruptor *probe with one of its *corrupted *probes.

The total number of *corruptor/corrupted *probe pairs reflects the amount of cross-hybridization in the microarray experiment.

### Output

**Figure 1:**
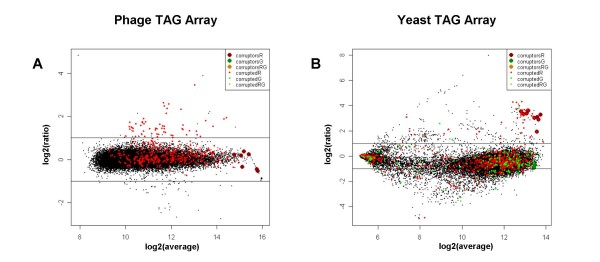
**Examples of cross-hybridization in two independent data sets**. Magnitude versus amplitude (MA) plot of two arrays from the "Phage TAG array" (A) and the "Yeast TAG array" (B) data sets. The x-axis represents the average log2 intensity of the two channels, and the y-axis represents the log2 ratio of channel1/channel2. Probes identified as *corruptors *or *corrupted *in the first (R), the second (G) and both (RG) channels are highlighted with the respective labels. Horizontal lines indicate 2-fold change (log2 ratio = -/+1).

Different types of outputs are generated as results of the analysis (see also Additional file 1):

1) A plot showing for each analyzed probe the p-value resulting from the Monte Carlo simulation

2) A list of the probes identified as *corruptors *and their related p-values

3) A list of the probes identified as *corrupted *and their related p-values

4) A plot showing the spatial distribution of *corruptors *and *corrupted *probes on the array

5) A plot showing the ratio versus average intensity values (*MA plot*) with *corruptors *and *corrupted *probes highlighted in colors (Figure 1).

### Performance

The analysis of a double channel Agilent 22K array (with the default parameters) takes about 8 min using a Pentium 4, 3 GHz, 1GB RAM

A step-by-step description on how to run an exemplary microarray analysis and the output figures is illustrated in supplemental materials (Additional file 1).

## Results

To validate the software and the related methodology, we have used the *CrossHybDetector *software to analyze independent microarray data sets obtained from two different array layouts, here referred as "Phage TAG array" and "Yeast TAG array". In both cases the oligonucleotide probes spotted on the arrays were representing artificially designed "DNA barcodes". These types of arrays are largely used in a variety of applications to monitor the respective abundances of DNA synthetic sequences ("barcodes") present in different samples [9,10].

The "Phage TAG array" dataset is composed of 76 previously reported microarray experiments carried out in our laboratory [11]. The array layout includes probes complementary to a repertoire of 20.736 synthetic DNA "barcode" sequences and was designed to analyze collection of phage clones tagged with short synthetic DNA sequences [11]. The "Yeast TAG array" dataset contains 135 microarray generated with in-house synthesized oligonucleotide arrays [12,13]. The array layout includes probes complementary to a collection of 11.986 different DNA barcodes. In this case, it was designed to profile the relative abundances of yeast strains from the Yeast Knockout (YKO) strain collection [12,13], tagged with short synthetic DNA sequences.

The results obtained by applying *CrossHybDetector *to each hybridization experiment of the two data sets (using a Monte Carlo p-value threshold equal to 0.01) are presented in Table 1. In both data sets *corruptor *and *corrupted *probes were identified. In the case of the "Phage TAG array" data set, after the adoption of more stringent conditions to the hybridization and washing protocols, the hybridization experiments affected by cross-hybridization problems decreased from 22 (out of an initial number of 152 hybridization experiments) to 5 (data not shown).

**Table 1:** Summary results of *CrossHybDetector*

	***Phage TAG Array***	***Yeast TAG Array***
Oligonucleotide probes spotted on the array	20736	11986
Arrays analyzed	76	135
Hybridization exps analyzed	152	270
Hybridization exps affected by cross-hybridization events	22	35
Probes identified as *corrupted *in more than 10% hybridization exps	277	940

The output plots in Figure 1 (panels A and B) show the results of the *CrossHybDetector *analysis in one experiment affected by cross-hybridization events in each of the two data sets. In panel A, it can be observed that many spots with high intensity values are identified as *corruptors *and affect a large part of the probes with a fold change greater than two, thus resulting as *corrupted*.

The complete set of output results can be generated by following the software guidelines provided as supplemental materials (Additional file 1).

To further validate the results, we separately run an additional analysis on the probes identified as *corrupted *in more than 10% of the experiments of each data set.

For each *corrupted *probe, the correlation with each of its respective *corruptors *in the different experiments was calculated across the entire data set.

**Figure 2:**
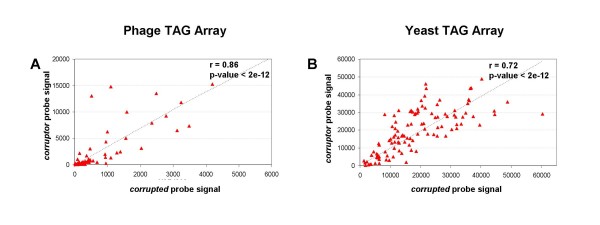
**Plots of the intensity of *corruptor *versus *corrupted *probes**. Correlation between the probe intensity values of two *corruptor/corrupted *pairs, respectively identified in each data set. Each point represents a different array hybridization experiment. The Pearson's correlation and the corresponding p-values are indicated.

The rationale for this analysis is that any statistically significant correlation observed between two probes of a *corruptor/corrupted *pair can only be ascribed to cross-hybridization effects as no functional relationship exist between the synthetic sequences monitored in the two experiment settings. Results are illustrated in Table 2. In both data sets we observed that the average Pearson's correlation for the whole set of *corruptor/corrupted *pairs was significantly higher than the average correlation obtained on an equal number of randomly selected probe pairs (10,000 Monte Carlo samplings, p-value < 0.0001). As an example, the correlation between the probes of two *corruptor/corrupted *pairs, respectively identified in each data set, is shown in Figure 2.

**Table 2:** Summary results of the probe pairs correlation analysis

	***Phage TAG Array***	***Yeast TAG Array***
*Corruptor/corrupted *probe pairs	3034	1048
Average Pearson's correlation	0.75	0.24
Monte Carlo Average Pearson's correlation (on 10,000 random pairs selections)	0.36	0.18
Monte Carlo p-value	<0.0001	<0.0001

These results represent independent confirmation that the probes identified as *corrupted *in individual experiments by the *CrossHybDetector *were affected by artifactual cross-hybridization effects.

## Discussion and conclusion

Other methods have been reported that address the problem of cross-hybridization in DNA microarray. Flikka et al. [14] developed a web-tool for the assessment of the reliability of hybridization signals in different array designs by comparing probe sequences against human, mouse and rat transcript collections. Gene candidates for cross-hybridization are selected on the basis of sequence similarity calculated using the BLAST algorithm [15]. As opposed to *CrossHybDetector*, this tool was not conceived as a quality control tool for array hybridization experiments and it does not take into account the hybridization signals derived from specific experiments. A different approach was used by Casneuf and collaborators [16]. Here probe sets affected by off-target hybridization were identified on the basis of positive correlations between sequence similarity and expression across a series of microarray experiments. This approach is similar to the correlation analysis we carried out to validate the results of *CrossHybDetector*. The most important difference between the two methodologies lies in the fact that while Casneuf's approach uses an entire data series to identify potential cross-hybridizing probes, *CrossHybDetector *focuses on individual hybridization array experiments.

In this respect, *CrossHybDetector *is mainly a quality control software for single hybridization experiments, conceptually similar to other published tools aiming at the monitoring of different quality parameters such as the 'geographical' bias, the spot replicate concordance, the two-channel correlation [17,18]. Other methods also exist, which are aimed at correcting the spot intensities with a model-based approach [6,7] and therefore with a different purpose than experimental quality control.

*CrossHybDetector *uses both the probe intensity values and the probe sequences to identify potentially 'corrupted' spots. As a consequence, cross-hybridization events that do not cause an intensity increase of the spots carrying similar probe sequences cannot be detected. This has to be considered as an intrinsic limitation due to the 'in-silico' nature of the method and does not affect its general utility.

*CrossHybDetector *can be applied to all arrays where for each individual probe its intensity signal is provided. As a consequence, it can not be applied to Affymetrix chips where the intensity values are associated to multiple probes (PM and MM probe sets). We envisage *CrossHybDetector *to be extremely useful to quality control experiments performed on customized microarrays. In these particular settings, microarrays are more prone to present issues related to suboptimal probe design and/or experimental conditions.

Moreover, being developed as a standard R package, *CrossHybDetector *is well suited to integrating into more complex quality control platforms and automated analysis workflows.

## Availability and requirements

**Project name: **CrossHybDetector

**Project home page: **

**Operating systems: **The crosshybDetector package can be installed on all the platforms supporting R. These include a wide variety of UNIX platforms, Windows and MacOS.

**Programming language: **R

**License: **LGPL

## Authors' contributions

PU conceived and implemented the method, wrote the code and drafted the manuscript. EDR contributed to the conceptualization of the method and wrote the final version of the manuscript. Both authors read and approved the final manuscript.

## Supplementary Material

Additional file 1 **Application of *CrossHybDetector *to a microarray experiment.** PDF file containing step-by-step instructions on how to run a complete analysis using the *CrossHybDetector *package. The document also includes all the plots produced by the package. Input files used in this example are available in Additional file 2. Click here for file

Additional file 2 **Input files used for *CrossHybDetector *exemplary analysis.** This ZIP archive include three files containing i) the array probe sequences, ii) the array spot intensities and array layout, iii) the spot type information (i.e. for each spot, whether it is "probe", "negative control", "spike-in"). Click here for file
