# Supplementary material for: CrossHybDetector: detection of cross-hybridization events in DNA microarray experiments
Source: BMC Bioinformatics. 2008 Nov 17;9:485. doi: 10.1186/1471-2105-9-485 (PMC2596149; doi:10.1186/1471-2105-9-485)
Supplement: Additional file 1 — Application of CrossHybDetector to a microarray experiment. PDF file containing step-by-step instructions on how to run a complete analysis using the CrossHybDetector package. The document also includes all the plots produced by the package. Input files used in this example are available in Additional file 2. [file 1471-2105-9-485-S1.doc]

**SUPPLEMENTARY INFORMATION**

**Application of *CrossHybDetector* to a microarray experiment**

The current version of CrossHybDetector supports double-channel array data only. A typical analysis consists of three main steps:

1. data preparation
2. computation of *p*‑values and identification of probes affected by cross-hybridization
3. generation of the output and plot files

A detailed description of each function used here is available in R using the help command:

> help(myfunction)

Before running the analysis, download and install the package from the Comprehensive R Archive Network (CRAN) at [http:/**/**cran**.**r-project.org/](http://cran.r-project.org/), then load the package. This will also load the necessary *methods* and *marray* packages.

*1. Data preparation*

The algorithm uses as input i) the array probe sequences, ii) the spot intensities and array layout, iii) the spot type information (i.e. for each spot, whether it is “probe”, “negative control”, “spike-in”). The information is contained into three separated text files (exemplary input files are submitted as supplemental materials –see Additional file 2).

The function read.delim is used to import the sequences from a text file (“sequences.txt” in the example) into a character vector:

> probeSeq <- read.delim(file="sequences.txt", as.is=TRUE)

Sequences can be extracted from the microarray annotation file describing the array layout (i.e. GAL file). When sequence are not proptly available and only their gene locations are provided, sequences can be retrieved using the getSequence function from the *biomaRt* R package (see the *biomaRt* documentation for additional details).

The intensity data and array layout information are read from a second file (“myArray.gpr” in the example) and included in an object of the R class *marrayRaw*. This file is normally generated by the image analysis software in the standard “GenePix” format.

> library(marray)

> raw <- read.GenePix( fnames = "myArray.gpr",

path = NULL,

name.Rf = "F1 Median",

name.Rb = "B1 Median",

name.Gf = "F2 Median",

name.Gb = "B2 Median",

name.W = "Flags",

sep = "\t"

)

The spot type information are read from a third text file (“mySpotTypes.txt” in the example) and added to the *marrayRaw* object:

> library(arrayQuality)

> myControlCode <- readcontrolCode(file="mySpotTypes.txt",

controlId="ID")

> maControls(raw)<- as.factor(maGenControls(maGnames(raw),

controlcode = myControlCode,

id = "ID")
 )

> table(maControls(raw))

An exemplary dataset of this type can be directly loaded from the *crosshybDetector* package:

> data(probeSeq)

> data(raw)

*2. Computation of p-values and identification of probes affected by cross-hybridization*

Once the input object is ready, the crosshyb function can be run to compute the probability of cross-hybridization. In the example shown here, the number of Monte Carlo simulations has been set to 10,000. The function creates a list of all the analyzed probes and their corresponding p-values:

crosshyb.out <- crosshyb(raw, probeSeq, numPermut=10000,

probeNameID="Name",

probes=c("probes","spike"),

delta=10

)

The smaller the calculated p-value the higher is the probability of the probe to be a “corruptor” (see algorithm description in the manuscript). The results of the analysis are shown in Figure 1 were probe intensity rank vs p-values are plotted using the function:

crosshybMCplot(crosshyb.out, pVal=0.01,

arrayName="myArray", doPlot=FALSE)

By using the exemplary data set 6 probes appeared to be “corruptors” on the red channel and 2 on the green channel, using 0.01 as p-value threshold.

The identifiers of the “corruptor” probes can be extracted by using the function:

badProbes <- extractBadProbes(crosshyb.out, pVal=0.01)


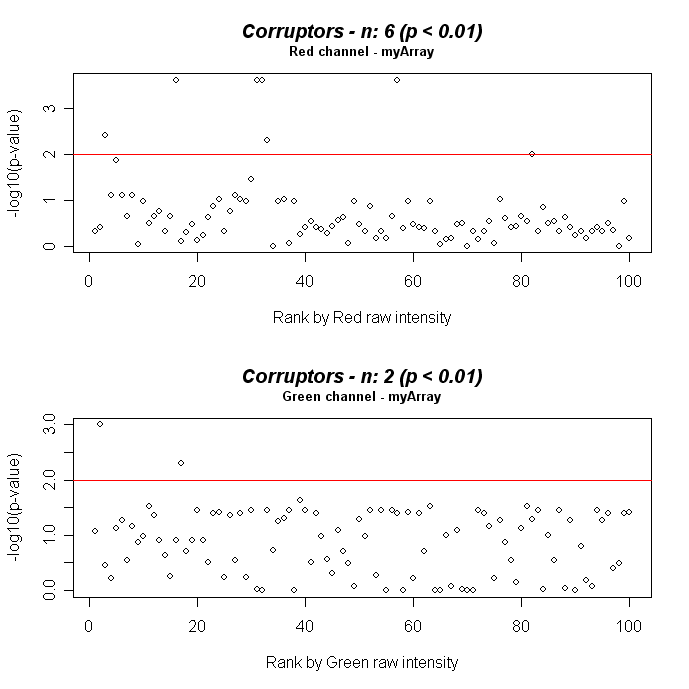


**Figure 1**. Output of crosshybMCplot function. This function draws two scatter plots, one for each channel, containing the *p*-value from Monte Carlo simulations ordered by probe intensity. Here the first 100 probes have been processed. The X and Y axis respectively represent the intensity rank and the –log10 of the Monte Carlo calculated *p-value*. On the Y axis

a red line corresponding to pVal=0.01 is shown. Probes above the red line (i.e. whose pvalue < pVal) are flagged as corruptors.

*3. Writing of output files*

In this final section, the plotting function crosshybImage has been used to create the image plots of the array with the spatial distribution of *corruptor* and *corrupted* probes (Figure 2). In our example we detected 237 (1.1% of the total number of probes) and 86 (0.4%) corrupted probes for red and green channels respectively:

# RED channel

crosshybImage(raw, plate = 1,

parent=badProbes$corruptorsR, children=badProbes$corruptedR,

arrayName="myArray",

channel="red", doPlot=FALSE)

# GREEN channel

crosshybImage(raw, plate = 1,

parent=badProbes$corruptorsG, children=badProbes$corruptedG,

arrayName="myArray",

channel="green", doPlot=FALSE)

The probe names and the corresponding raw intensities of the probes identified as “corruptors” or “corrupted” are written into tab-delimited files:

crosshyb2xls(raw, array=1,

parent=badProbes$corruptorsR,

children=badProbes$corruptedR,

arrayName="myArray",

channel="red", probeNameID="Name")

crosshyb2xls(raw, array=1,

parent=badProbes$corruptorsG,

children=badProbes$corruptedG,

arrayName="myArray",

channel="green", probeNameID="Name")

In addition, a MA-plot of normalized ratios can be created, displaying the log-ratios (M) and the log-intensities (A) on Y and the X axis respectively (Figure 3). *Corruptor* and *corrupted* probes are highlighted in colors:

# Loess normalization using only probes

library(marray)

norm <- maNorm(raw, norm="l",

subset=maControls(raw) == "probes")

crosshybMAplot(m=maM(norm[,1]),

a=maA(norm[,1]),

subset=maControls(raw) %in%

c("probes","spike"),

badProbes=badProbes,

arrayName="myarray",

doPlot=FALSE)


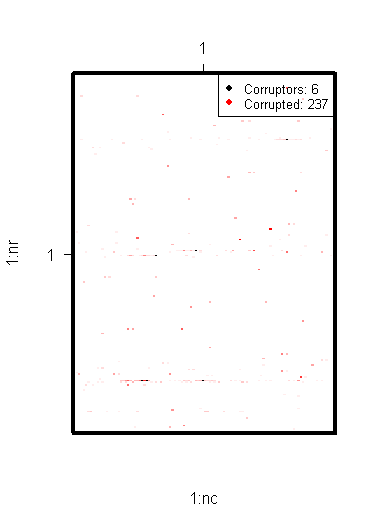

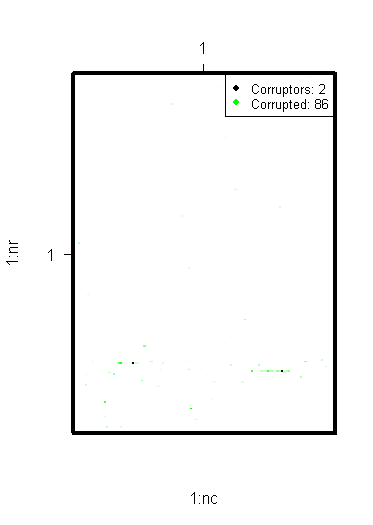


**Figure 2**. Output of crosshybImage function. This function creates an image with the spatial distribution of the *corruptor* and *corrupted* probes on the array. The color intensity of *corrupted* probes is proportional to the intensity value. The presence of *corruptors* on the same array row generates the occurrence of red and green segments in the plot. This effect results from the particular chip design, in which similar probes were spotted on close positions on the array.

**
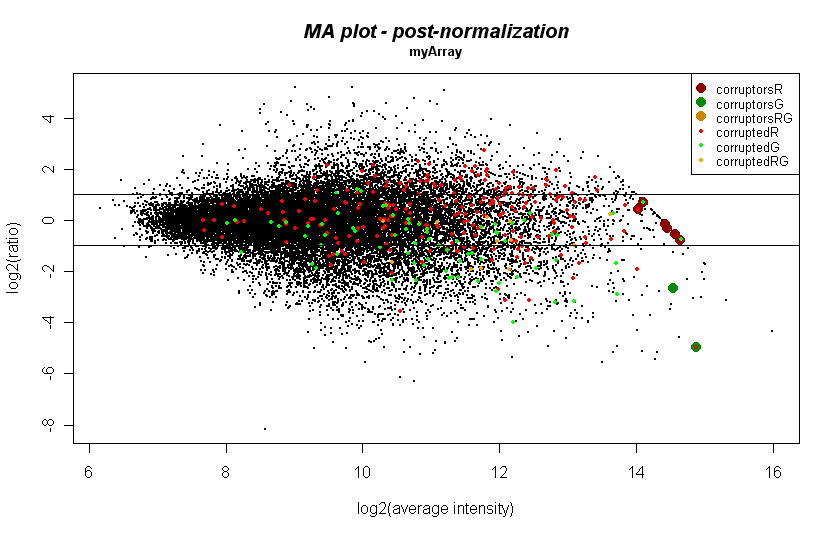
**

**Figure 3**. Output of crosshybMAplot function. This function draws a plot of log-intensity ratios (M-values) versus log-intensity averages (A-values). Probes identified as potentially *corruptors* and *corrupted* in red (R), green (G) and both channels (RG) are highlighted. Here many high intensity spots are flagged as *corruptors* and affect a part of up-regulated probes. Horizontal lines indicate 2-fold change (log2 ratio = -/+1).

To assess the effect of the different metrics that can be used to measure the pairwise sequence similarity, we compared the results obtained with the 'Smith-Waterman score' (default metric) and the 'percentage of sequence identity'. The p-values from 10,000 random Monte Carlo simulation for 50 probes are shown in Figure 4. Overall the two metrics produced the same results; the small differences observed in few cases reflect the stochastic nature of the Monte Carlo analysis


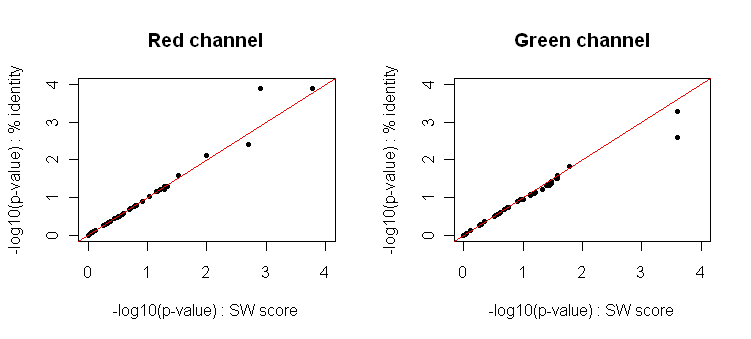


**Figure 4**. Comparison of p-values from Monte Carlo simulations obtained using two different metrics of pairwise sequence similarity: Smith-Waterman score and percentage of sequence identity. The scatter shows the p–values for 50 probes analyzed on both channels.

**Data availability**

Datasets and example code are included in the *crosshybDetector* package freely available at http://cran.r-project.org/.
